# Effect of Multiple Sclerosis Cerebrospinal Fluid and Oligodendroglia Cell Line Environment on Human Wharton’s Jelly Mesenchymal Stem Cells Secretome

**DOI:** 10.3390/ijms23042177

**Published:** 2022-02-16

**Authors:** Karolina Salwierak-Głośna, Paweł Piątek, Małgorzata Domowicz, Mariola Świderek-Matysiak

**Affiliations:** 1 Department of Neurology, Medical University of Lodz, 90-419 Lodz, Poland; karolina.salwierak-glosna@umed.lodz.pl (K.S.-G.); pawel.piatek@umed.lodz.pl (P.P.); malgorzata.domowicz@umed.lodz.pl (M.D.); 2 Department of Immunogenetics, Medical University of Lodz, 90-419 Lodz, Poland

**Keywords:** multiple sclerosis, mesenchymal stem cells, secretome, cerebrospinal fluid, oligodendrocytes

## Abstract

Multiple sclerosis (MS) is a neurological disorder of autoimmune aetiology. Experimental therapies with the use of mesenchymal stem cells (MSCs) have emerged as a response to the unmet need for new treatment options. The unique immunomodulatory features of stem cells obtained from Wharton’s jelly (WJ-MSCs) make them an interesting research and therapeutic model. Most WJ-MSCs transplants for multiple sclerosis use intrathecal administration. We studied the effect of cerebrospinal fluid (CSF) obtained from MS patients on the secretory activity of WJ-MSCs and broaden this observation with WJ-MSCs interactions with human oligodendroglia cell line (OLs). Analysis of the WJ-MSCs secretory activity with use of Bio-Plex Pro™ Human Cytokine confirmed significant and diverse immunomodulatory potential. Our data reveal rich WJ-MSCs secretome with markedly increased levels of IL-6, IL-8, IP-10 and MCP-1 synthesis and a favourable profile of growth factors. The addition of MS CSF to the WJ-MSCs culture caused depletion of most proteins measured, only IL-12, RANTES and GM-CSF levels were increased. Most cytokines and chemokines decreased their concentrations in WJ-MSCs co-cultured with OLs, only eotaxin and RANTES levels were slightly increased. These results emphasize the spectrum of the immunomodulatory properties of WJ-MSCs and show how those effects can be modulated depending on the transplantation milieu.

## 1. Introduction

Multiple sclerosis (MS) is a neurological disorder with complex pathology, such as inflammation, demyelination, axonal loss, astrocytosis and microglia activation [1]. Currently available therapies mostly use immunomodulatory drugs, which are effective, but especially so in the early stages of MS, when the inflammatory process in the central nervous system (CNS) is dominant. The problematic question is targeting the neurodegenerative process during the MS course in the relapsing and progressive form of the disease [2].

Cell-based therapy using transplantation of mesenchymal stem cells (MSCs) came as a novel approach to that issue, sparking interest and hope [3]. Initial data from experiments using MSCs treatment in animal models of MS—experimental autoimmune encephalomyelitis (EAE)—were promising, showing reduction of inflammatory infiltrates and demyelination areas, stimulating oligodendrogenesis, and increased brain derived neurotrophic factor (BDNF) expression [4,5,6,7]. The remyelination effect achieved in animal experiments was not extrapolated in humans by straight intracranial transplantation of oligodendrocyte progenitor cells (OPCs) or neural stem cells (NSCs) [8,9]. Transplantation of NSCs as a source of cytokines and neurotrophins for a local neuroprotective function, but also an immunosuppressive response, is a promising therapeutic strategy [10,11].

An interesting issue is how the transplantation environment influences the repertoire of immune and trophic factors secreted by MSCs. Karussis et al. have indicated no differences between outcomes in intravenous (IV) and intrathecal (IT) routes of MSCs delivery in MS [12]; intrathecal administration is an encouraging therapeutic option with a considerable efficacy and safety profile [12]. In addition, Petrou et al. showed data for a slightly lower percentage of patients with treatment failure after intrathecal transplant of MSC versus those who were given MSC intravenously [13]. Another publication by Uccelli reported little survival of MSCs and poor distribution to CNS circulation after intravenous transplantation [14]. 

In the light of growing evidence for the healthy adult CSF promotion of predominantly astroglial differentiation of cultured NSCs and to a lesser extent oligodendroglial and neuronal differentiation [15], the study of MSCs-CSF interaction gains importance. Moreover, the observation that the use of different sources of CSF may initiate promotion and differentiation of specific cell lines was confirmed in the case of the CSF from progressive MS patients; inhibition of NSCs proliferation and promotion of neuronal and oligodendroglial differentiation were shown [16]. Although initially, most attention was paid to the stromal progenitors of mesodermal cells, bone marrow-derived mesenchymal stromal cells (BM-MSCs) and alternatively adipose mesenchymal stromal cells (AMSCs), soon the potential of MSCs obtained from perinatal tissues was also noticed in the field of cell therapies [17]. Cord blood and cord tissue was shown to be an excellent source of MSCs populations: Wharton’s jelly Mesenchymal Stem Cells (WJ-MSCs) and Human Umbilical Cord Perivascular Cells (HUCPVCs). Next to the characteristics of all MSCs types: adherence to plastic, expression of CD105, CD73, CD90 membrane antigens and the ability for multilineage differentiation under adequate conditions, WJ-MSCs display unique properties [18]. They possess fibroblast shape in culture, express CD29, CD44, CD51, SH2, SH3, and are negative for CD34, CD45 [19], and manifest immunomodulatory activity. Regarding their immunoregulatory functions, the secretory activity of WJ-MSCs deserves special attention, which is currently assigned a prominent role in interacting with the immune system. WJ-MSCs have shown ability to release cytokines and chemokines: IL-10, TGF-β, IL-6, FGF, VEGF [20,21]. The published experience of the clinical use of WJ-MSCs is modest but promising. A randomized double-blinded, placebo-controlled study, conducted with relapsing-remitting MS and secondary progressive MS patients, showed that the use of placenta derived mesenchymal cells was effective and safe [22]. The exact mechanisms of the WJ-MSCs action remained unclear. Liu et al. showed a shift of Th1 to Th2, and reduced demyelination caused by transplantation of WJ-MSCs [23]. Subsequent experiments with the use of WJ-MSCs licensed in vitro with highly pro-inflammatory cytokines (IFN-γ, Il-6, TNFα) showed the importance of inflammatory milieu for immunomodulatory activity of WJ-MSCs on-site [24]. Systemically infused WJ-MSCs ameliorated the EAE, this protective effect was related to the reduction of autoantigen-induced T-cell proliferation. Surprisingly, licensed WJ-MSCs did not ameliorate the EAE with a fast rejection due to increased immunogenicity. 

In this study we asked the questions, what are the effects of the adult human CSF obtained from MS patients and healthy controls on the secretory activity of WJ-MSCs, and how does the WJ-MSCs interaction with the human oligodendroglia change secretome of stem cells. Therapy based on MSCs is a promising treatment option in inflammatory and neurodegenerative diseases of the central nervous system, but the use of MSCs as a product for creating a new drug instead of cell transplantation might be more effective and predictable.

## 2. Results

### 2.1. Secretome of WJ-MSCs Conditioned with MS and Control CSF

**Figure 1:**
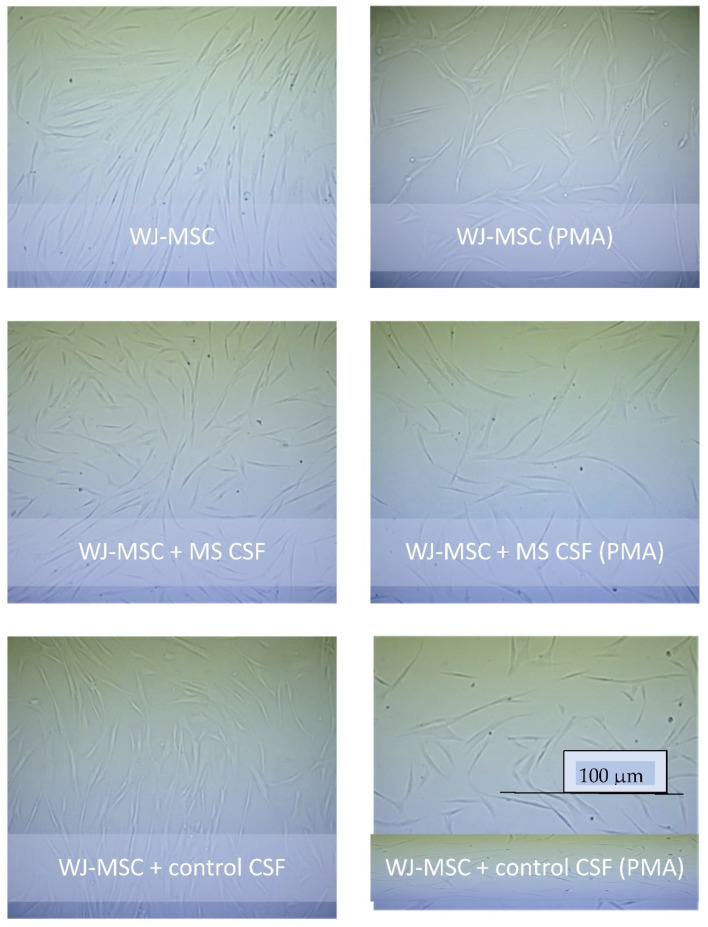
Microscopic features of cell survival and growth of WJ-MSCs in medium and PMA conditions and with MS CSF versus control CSF addition. Scale bar: 100 μm.

**Figure 2:**
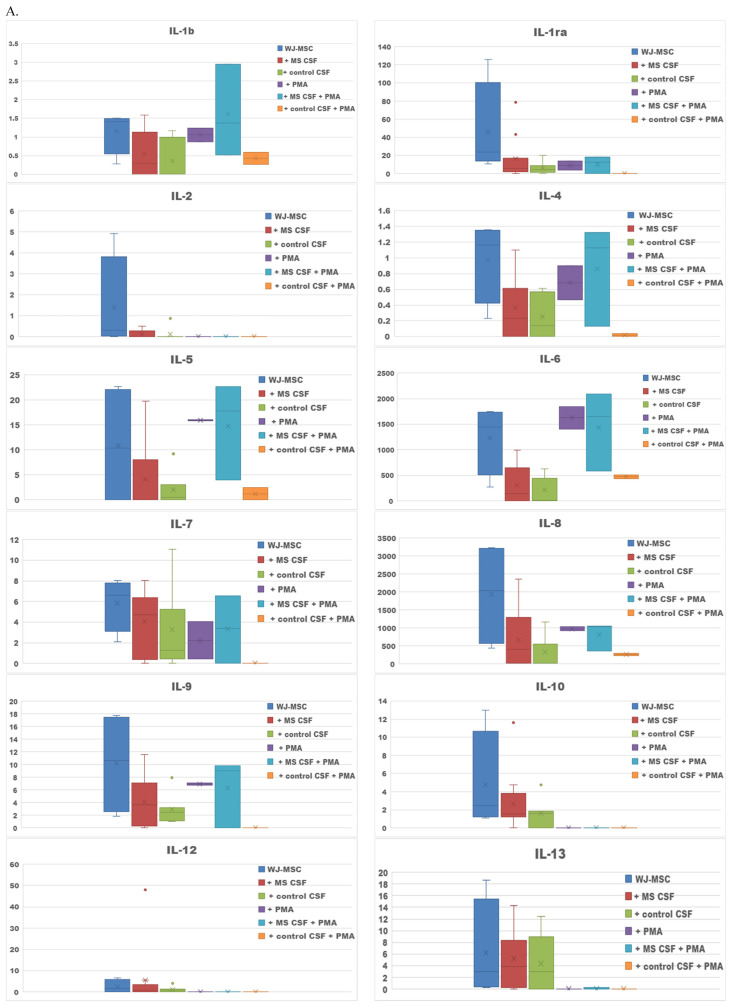

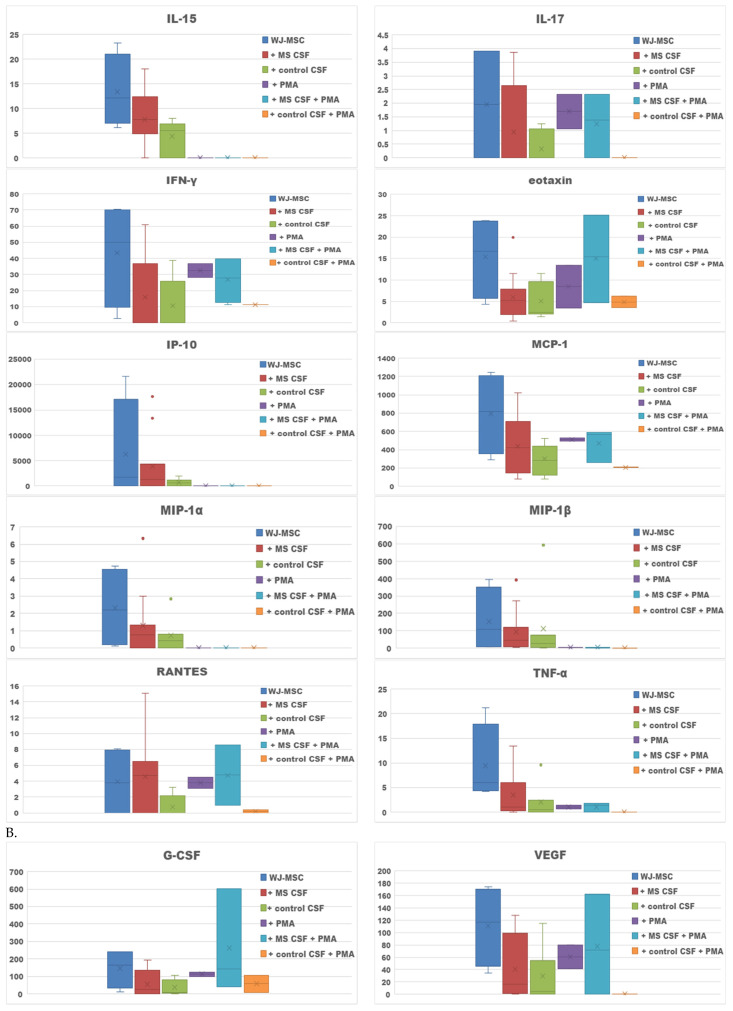

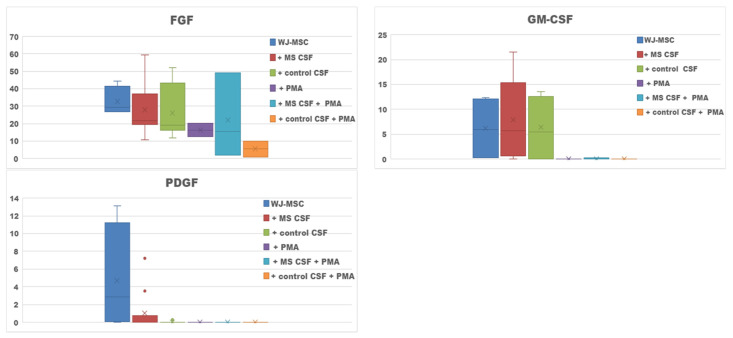
The effect of the WJ-MSCs culture conditioned with MS CSF and the control CSF conducted with the addition of phorbol 12-myristate 13 acetate (PMA). The average concentration of secreted cytokines, chemokines (**A**) and growth factors (**B**) given in pg/mL as means ± SD from 4 samples for WJ-MSCs, 11 for WJ-MSCs + MS CSFs and 7 for WJ-MSCs + control CSFs, from 2 experiments for WJ-MSCs with PMA, 3 for WJ-MSCs + MS CSF with PMA and 2 for WJ-MSCs + control CSF with PMA. Statistically significant decreases in secretion (*p* < 0.05) were observed for Il-4, Il-6, Il-8, eotaxin and VEGF for the WJ-MSCs culture compared to the WJ-MSCs with MS CSF; Il-1b, Il-4, Il-6, Il-8, Il-9, Il-15, eotaxin, IFNγ, MCP-1, G-CSF and VEGF for the WJ-MSCs culture compared to WJ-MSCs with the control CSF.

The survival properties of WJ-MSCs in different culture conditions are shown as microscopic images (Figure 1). Secretome obtained from the WJ-MSCs cultures turned out to be rich in cytokines, representatives for both—humoral immunity: interleukins IL-4, IL-5, IL-13; and cell-mediated immunity: IL-2, IL-12, IFN-γ (Figure 2A). Among the secreted factors, the highest concentrations were noted for IL-6, IL-8, IP-10 and MCP-1. Primarily IL-6, but also IL-1, TNF-α, IFN-γ of the proinflammatory cytokines were obtained in secretome. The cytokines involved in the anti-inflammatory response, IL-1ra, IL-4, IL-10, and IL13, were also shown. 

The addition of MS CSF to the WJ-MSC culture depleted most of the proteins measured: IL-1, IL-1ra, IL-2, IL-4, IL-5, IL-6, IL-7, IL-8, IL-9, IL-10, IL-13, IL-15, IL-17, eotaxin, IFN-γ, IP-10, MCP-1, MIP-1α, MIP-1β, TNF-α (Figure 2A). The average concentrations of the two factors: IL-12 and RANTES were slightly increased in WJ-MSCs conditioned with MS CSF compared to the WJ-MSCs culture. The addition of the control CSF caused even lower concentrations of cytokines compared to samples with WJ-MSCs and WJ-MSCs conditioned with MS CSF. No statistically significant differences were observed between cultures of WJ-MSCs with MS CSF and the control CSF. We also measured concentrations of five growth factors: G-CSF, VEGF, FGF, GM-CSF, PDGF, which were the most abundant in the secretome of WJ-MSCs; the highest average levels were noted for G-CSF and VEGF. The addition of MS and control CSF caused decreased levels of G-CSF, VEGF, FGF and PDGF. Only for GM-CSF, was the average concentration higher in WJ-MSCs conditioned with MS CSF, compared to the WJ-MSCs culture (Figure 2B). 

The aim of PMA use in our study was to cause maturation of the oligodendrocytes precursor MO3.13 cell line into oligodendrocytes (OLs) during the next experimental step. PMA might also promote differentiation of stem cells; this forced introduction of PMA as a variable could also have a potential impact on secretory activity of WJ-MSCs. In the case of the WJ-MSCs culture conducted with PMA, we noted an increase of IL-5 and IL-6 compared to pure WJ-MSC culture. The concentrations of most measured factors decreased compared to the pure WJ-MSCs culture; several factors were not even detected. The culture of WJ-MSCs + MS CSF with PMA addition proved to have more abundant IL-1b, IL-1ra, IL-4, IL-5, IL-6, IL-7, IL-8, IL-9, IL-17, IFN-γ, eotaxin, MCP-1, RANTES compared to the WJ-MSCs + MS CSF culture. In the case of WJ-MSCs incubated with the control CSF and PMA, we noted increased levels of IL-1b, IL-6, IL-8, IFN-γ, eotaxin and RANTES compared to the pure culture of WJMSCs and the control CSF (Figure 2A). 

The culture supplemented with PMA also resulted in changed concentrations of the growth factors. In the WJ-MSCs culture with PMA, we noted depletion of each secreted trophic factor compared to the pure WJ-MSCs culture. The tendency to lower concentrations of trophic factors was also observed for the WJ-MSCs culture with control CSF and PMA—the only exception was G-CSF. In the WJ-MSCs culture with MS, CSF and PMA, depleted levels of GM-CSF and PDGF, and increased levels of G-CSF and VEGF, were noted compared to the WJ-MSCs culture with MS CSF (Figure 2B) (Table 1).

### 2.2. Effect of Oligodendrocytes Precursor Cell Line Co-Culture on WJ-MSC Secretome

**Figure 3:**
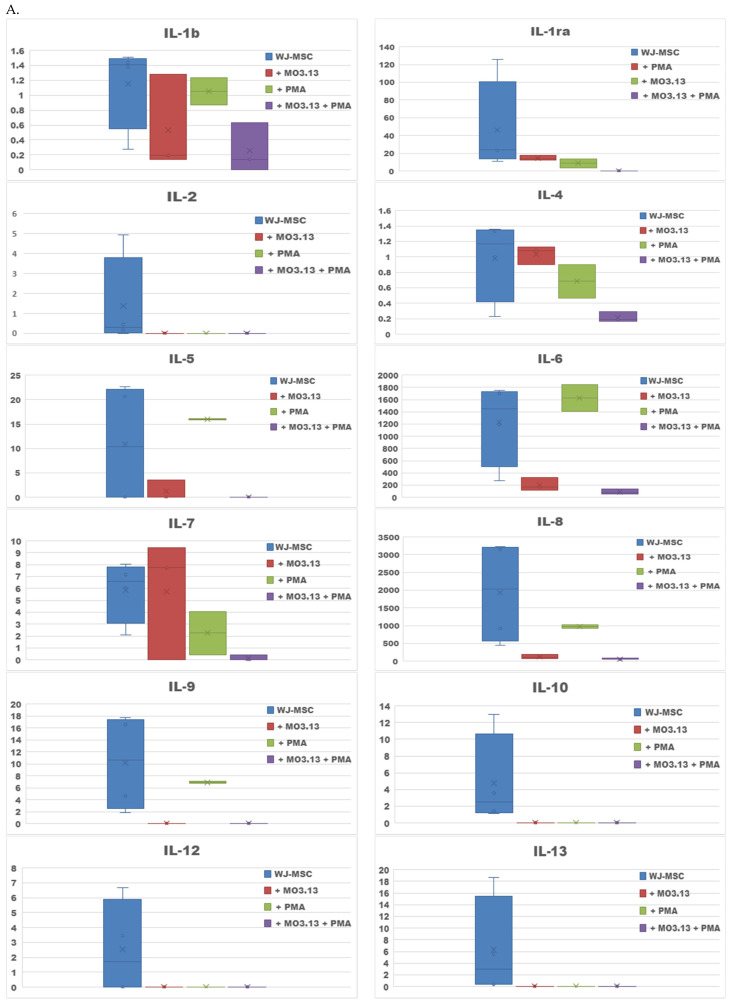

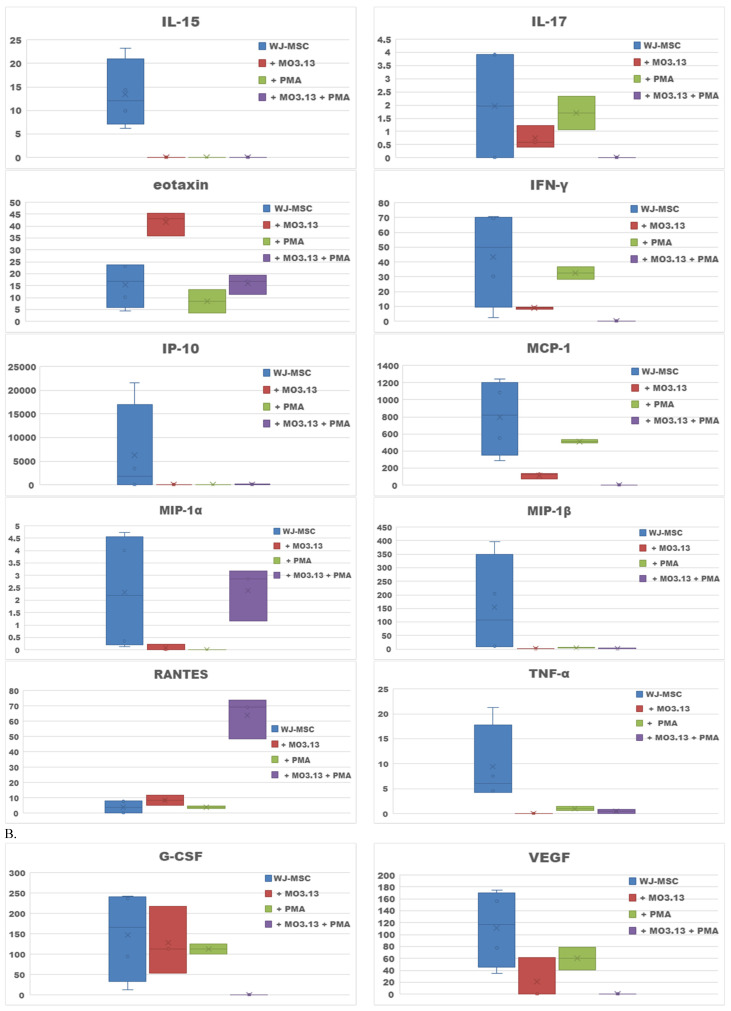

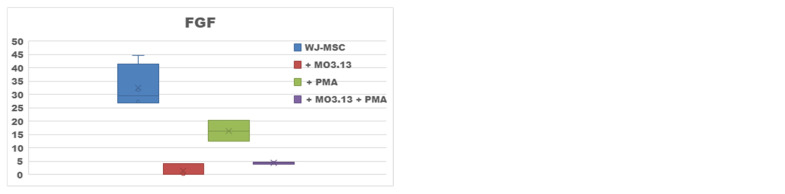
The average concentrations of cytokines and chemokines (**A**) and growth factors (**B**) secreted in co-cultures of WJ-MSCs and MO3.13 cell line (pure and with addition of PMA) compared to the WJ-MSCs culture. Data are given in pg/mL as means ± SD from 4 samples for WJ-MSCs, 3 samples for WJ-MSCs + MO3.13, 2 samples for WJ-MSCs with PMA, 3 samples for WJ-MSCs + MO3.13 with PMA.

The next aim of our study was to estimate how the inherence of the oligodendrocytic cell line MO3.13 affected the secretory profile of the WJ-MSC culture. In this case we analysed secretomes of WJ-MSCs seeded simultaneously with MO3.13 (Figure 3A). Referring to the results obtained in the previous part of the study, we noted that the secretory profile of WJ-MSCs co-cultured with MO3.13 was more modest than the secretome of the WJ-MSCs. The average concentrations of most cytokines and chemokines decreased compared to the WJ-MSC culture (IL-1b, IL-1ra, IL-4, IL-5, IL-6, Il-7, IL-8, IL-17, IFN-γ, MCP-1, MIP-α, FGF, G-CSF, VEGF), or were not detected (IL-2, IL-9, IL-10, IL-12, IL-13, IL-15, MIP-1β, TNF-α, GM-CSF, PDGF) (Figure 3B). Only two chemokines—eotaxin and RANTES—have slightly increased average concentrations compared to the WJ-MSC culture.

In the co-culture of WJ-MSCs and MO3.13 conducted with the addition of PMA, we found the presence of Il-1b, IL-4, IL-6, IL-7, IL-8, eotaxin, IFN-γ, MCP-1, MIP-1α, RANTES, TNF-α and only one growth factor—FGF. Only the levels of MIP-1α and RANTES were higher in WJ-MSCs + MO3.13+ PMA co-culture compared to the WJ-MSCs + MO3.13 co-culture (Table 1).

## 3. Discussion

Intrathecal administration of WJ-MSCs as an attempted MS therapy generates new possibilities and concerns. The initial approach to this therapy, that emphasized the potential of WJ-MSCs, relied on their multilineage differentiation. We now understand that secretory activity, that creates a special immunomodulatory milieu and promotes a regenerative process, is an attractive feature for potential therapeutic application. Intrathecally administrated MSCs may be transferred along with the circulation of CSF to fluid spaces within the brain. Current reports show that interactions between MSCs and neurogenic regions in the brain are crucial for neuromodulation in MS [25]. The impact of cerebrospinal fluid on MSCs appears to be an important modulator and remains unexplored. This study was designed to assess the immunomodulatory potential of WJ-MSCs, alone and in contact with adult human CSF derived from MS patients and healthy controls. So far, the observations of the interaction between CSF and neural stem cells have been successfully conducted with cells of embryonic [15,26,27] or of adult origin (BM-MSCs) [28,29]. To our knowledge, this is the first study with the use of human WJ-MSCs and adult human CSFs collected from MS patients as well as healthy controls, assessing the humoral response in such a wide spectrum.

The obtained results of the cytokine secretion profile of WJ-MSCs suggest their immunomodulatory potential with the presence of key representatives of both Th1 and Th2 response. The highest levels were reached by IL-6, IL-8, IP-10 and MCP-1. IL-6 is a pleiotropic and highly proinflammatory factor, playing a considerable role in MS pathology, especially connected with astrocytic secretion. Previous data about estimating the effect of IL-6 on MSCs are contradictory, however most revealed no substantial effect on cell viability and migration [30,31,32,33,34,35]. Moreover, Hagman et al. showed increased secretion of IL-10 by Il-6 induced NPCs, which may result in building a pro- and anti- inflammatory balance, but also leads to a reduction of neuronal differentiation and ultimately impairs neurogenesis. IL-6 is also known for impairing astroglial activation, and in consequence, reducing OLs differentiation [36]. Additionally, IL-6 plays a role in oxidative stress reduction by secreting VEGF [30]. High levels of IL-6 secreted by WJ-MSC in the context of MS pathology must be assessed very carefully. Increased levels of IL-6 and IL-8 may result in recruitment and activation of proinflammatory cells in the brain [36]. The function of IL-8 is related to leukocyte attraction and infiltration (mainly neutrophils) at the site of inflammation. In MS patients, IL-8 expression is associated with disease activity, exhibiting the highest levels in CSF during relapses [37,38]. Moreover, a significant correlation between severe disability in patients with MS and elevated levels of IL-8 in the CSF was recently shown [39].

The chemokines MCP-1 and IP-10, that were determined to be in substantial concentrations in the WJ-MSCs culture, are addressing an important immunoregulatory role in MS by influencing cell proliferation, survival and regulating cell migration (especially monocytes and macrophages) at the site of inflammation [32,40,41]. In the study with adult neural stem cells, CCL-2 was shown to be a chemotactic factor for NSCs as well and that effect was enhanced in the environment of astrocytes induced by TNF-α supplemented supernatants [42]. In animal models of MS, targeting MCP-1 signalling has proven crucial for further inflammatory detriment [43,44,45]. On the other hand, MCP-1 may also induce Th2 polarization in MS patients; a decrease in the MCP-1 level is linked with the elevation of levels of inflammatory factors in the CSF during a relapse and may precede the emergence of clinical symptoms [46]. On the other hand, there is an increased MCP-1 level after treatment with methylprednisolone [47,48]. Analysis of the CSF biomarker in patients after three intrathecal injections of MSCs-derived neural progenitors, revealed a decrease in MCP-1 and an increase in interleukin 8 levels, that confirmed the immunoregulatory properties of MSCs [49].

As we presumed, the composition of the supernatants obtained after WJ-MSCs were incubated with CSF differs from those of WJ-MSCs incubated in medium. We obtained reduced levels of most cytokines, chemokines and growth factors in the WJ-MSCs culture with both MS and control CSF. The only exceptions were IL-12 and RANTES for MS CSF, and GM-CSF in both MS and control CSF. At the same time, good survival of the WJ-MSCs was observed under both MS and control CSF conditions under microscopy. The CSF is a natural medium for MSCs; a variety of studies showed that CSF promotes survival of neuronal cells of embryonic origin [26]. Moreover, it has been recently observed that conditioning with CSF can be considered as one of a variety of protocols for obtaining NSCs from MSCs of diverse origin, as also is the case of embryonic MSCs. Ge et al. obtained NSCs from both bone marrow and cord blood—derived stem cells, after human adult CSF induction; they observed the first morphological changes after 24 h post-induction and astrocytes-like cells after 7 days [50]. Our study was completed 48 h after induction, this period may be too short to capture visible morphological changes in cells but considering microscopic features of good survival of WJ-MSCs in our study, differentiation is the most reasonable cause of the global decrease in the WJ-MSCs secretory activity. Other authors have reported that depending on the type of used CSF (adult, embryonic, healthy, MS or induced by various factors e.g., chemical, growth factors, injury) and the MSC origin, there were slight differences between fractions of obtained neuron-like cells. Most studies with human embryonic stem cells and healthy human CSF demonstrated predominantly glial differentiation [15,51,52]. One study with use of CSF from progressive MS patients and human embryonic-derived NPCs (ENStem-A) showed their increased differentiation towards neuronal and oligodendroglial cells. In our study, we observed a slight decrease of most secreted cytokines, chemokines and growth factors in samples conditioned with MS CSF, compared to the control CSF.

The increased levels of IL-12, RANTES and GM-CSF after conditioning the WJ-MSCs with MS CSF may have contributed to an adverse environment promoting the immune response in MS. However, in the light of reports on EAE, IL-12 and RANTES may play an alternative role for the development of MS [53]. Nevertheless, the evidence for GM-CSF participation in neuroinflammatory processes remains undoubted and was sealed with the trial of human antibody to GM-CSF, however in our study GM-CSF was present in a relatively low concentration.

Secretome of the WJ-MSCs culture was also rich in trophic factors, together we measured the levels of five agents: FGF, VEGF, G-CSF, GM-CSF, PDGF. The highest average levels were assigned to G-CSF and VEGF and both were several times less in cultures with both MS and control CSFs. That finding emphasized the inhibiting role of the WJ-MSCs—CSF interactions on the secretory activity of WJ-MSCs. This may be crucial for explanation of the trophic functions of undifferentiated MCSs and clinical outcomes after cell transplantation. The proposed mechanism of CSF-induced differentiation of MSCs into nerve cells is that the microenvironment within the brain, or the spinal cord, provides the necessary factors (still unknown) for the induced differentiation of MSCs. Our study showed that CSF itself significantly changed the secretory activity of the MSCs and thus also influenced the microenvironment.

The part of the experiment analysing the effect of the oligodendrocytes on WJ-MSCs showed a strong inhibition of secretory activity. Secretome obtained from co-cultures of WJ-MSCs and MO3.13, especially induced to maturation by PMA, were significantly depleted comparing to supernatants from the WJ-MSC culture conducted separately. Basically, the only cytokines present in significant amounts were IL-6, IL-8, MCP-1, eotaxin and RANTES. Noteworthy, in co-cultures of WJ-MSCs and MO3.13, we found three measured growth factors were positive, respectively: G-CSF, VEGF, FGF. The average concentrations of trophic factors were lower than those obtained from the WJ-MSCs cultures. The co-culture of oligodendrocytes had increased secretion of chemokines: eotaxin and RANTES. After PMA addition, as a maturation factor for the MO3.13 oligodendrocytic cell line, the expression level of the chemokines RANTES and MIP-1α were even enhanced. There are limited data on the direct interaction between human stem cells and oligodendroglia. A recently published study by Joerger-Messerli provided evidence that human extracellular vesicles derived from WJ-MSCs interacted with the MO3.13 cell line and accelerated their maturation after 5 days of co-culture [54]. In our study, we observed secretion of cytokines/chemokines/growth factors after 2 days of cell-to-cell contact of WJ-MSCs and MO3.13 and showed significant inhibition of MSCs secretory activity, which might be related to initiation of differentiation of MSCs. For therapeutic use, precisely prepared “drugs” like MSC secreted factors or vesicles, are a safer option for the future than using cells [55]. Even in the case of conditioning of stem cells culture with CSF, a novel approach is proposed to replace endogenous potentially pathogenic CSF by artificial CSF enriched with MSCs secreted factors. The treatment of EAE with MSC-secretions enriched with artificial CSF caused amelioration of the clinical symptoms [56]. Our results indicated that conditioning of MSCs with healthy, or multiple sclerosis CSF, might change the Secretome of MSCs and its immunomodulatory potential; furthermore, transplantation of MSCs to patients with different/unknown inflammatory status might promote a response impossible to foresee.

## 4. Materials and Methods

### 4.1. Cell Cultures

Human WJ-MSCs were acquired from the Polish Bank of Stem Cells (Warsaw, Poland), seeded on T175 Nunc flasks (Nunc, Thermo Scientific, Rostilde, Denmark) coated by 20 µg/mL fibronectin and incubated in DMEM/F-12 medium supplemented with 10% foetal bovine serum (FBS), 1% penicillin-streptomycin solution and enriched with 5 ng/mL EGF-epidermal growth factor (Gibco, Thermo Fisher Scientific, Darmstadt, Germany) and 5 ng/mL bFGF- fibroblast growth factor (Gibco, Thermo Fisher Scientific, Darmstadt, Germany).

Simultaneously, the human glia (oligodendrocytic) hybrid cell line (MO3.13) (Tebu-bio, Le Perray En Yvelines, France) was cultured in T75 Nunc flasks in DMEM-high-glucose medium supplemented with 10% FBS 1% penicillin-streptomycin solution. Both cultures were maintained at 37 °C with 5% CO_2_ in a humidified atmosphere and expanded for passages with use of trypsin-EDTA solution, 2 times in the case of the WJMSCs and 5 times for the MO3.13 cell line. The MO3.13 model was conducted with DMEM-high glucose serum supplemented with 1% penicillin-streptomycin solution, EGF and bFGF with the lack of FBS. The WJ-MSCs were cultured in DMEM/F-12 with the addition of 1% penicillin-streptomycin solution without FBS. The experiments were conducted on six-well plates, with both types of cells seeded with a density of 1 × 10^6^ per well. All samples had their counterparts treated with an addition of phorbol 12-myristate 13 acetate (PMA) (0.1 mM) as a factor conditioning oligodendroglia maturation. All reagents used for culturing were purchased from Sigma-Aldrich. The WJ-MSCs morphology and survival were analysed by imaging in DIC microscopy (Zeiss Axiovert 200 inverse microscope with a Zeiss Plan-apochromat 63×/1.43 differential interference contrast objective).

### 4.2. WJ-MSCs Culture Conditioning with MS and Healthy Control Cerebrospinal Fluid

The next step was incubating the WJ-MSCs with MS and healthy control CSF. The WJ-MSCs were seeded on six-well plates with a density of 1 × 10^6^ per hole. After 24-h incubation, 5% CSF suspended in the DMEM/F-12 medium (5 ng/mL EGF, 5 ng/mL bFGF, 1% penicillin-streptomycin without FBS) was added to each hole. After the next 24 h, we changed the medium to DMEM/F-12 without EGF and bFGF. Cultures were terminated after 24 h, supernatants were collected and secured by freezing at 80 °C for further analysis.

Cerebrospinal fluids were collected from patients hospitalized in the Department of Neurology, Medical University of Lodz. Lumbar punctures were performed as a part of diagnostic procedure. Patients were selected on the random basis. All MS patients were in remission, at least three months after relapse and steroids treatment. The experiment was approved by Medical University of Lodz Ethics Committee (RNN/218/18/KE).

### 4.3. Human Cytokine Multiple Profiling Assay in WJ-MSCs Cultures

The culture supernatants were collected and analysed for content of cytokines, chemokines and growth factors secreted during the experiment. Samples were obtained from the following cultures: WJ-MSCs; WJ-MSCs + MS CSF; WJ-MSCs + control CSF; WJ-MSCs + MO3.13; WJ-MSCs + MO3.13 + PMA. Cytokine/chemokine/growth factors concentrations in supernatants were measured using Bio-Plex Pro™ Human Cytokine Assays (Bio-Rad Laboratories, Hercules, CA, USA). We used a 27-plex assay to measure levels of interleukin 1 beta (IL-1β), receptor for interleukin 1 (IL-1ra), interleukin 2 (IL-2), interleukin 4 (IL-4), interleukin 5 (IL-5), interleukin 6 (IL-6), interleukin 7 (IL-7), interleukin 8 (IL-8), interleukin 9 (IL-9), interleukin 10 (IL-10), interleukin 12 (IL-12), interleukin 13 (IL-13), interleukin 17 (IL-17), interferon gamma (IFN-γ), interferon gamma-induced protein 10 (IP-10), monocyte chemoattractant protein 1 (MCP-1), macrophage inflammatory protein 1 alfa (MIP-1α), macrophage inflammatory protein 1 beta (MIP-1β), tumour necrosis factor alfa (TNF-α), regulated on activation, normal T cell expressed and secreted (RANTES), eotaxin, fibroblast growth factor (FGF), platelet derived growth factor (PDGF), vascular endothelial growth factor (VEGF), granulocyte colony-stimulating factor (G-CSF), granulocyte-macrophage colony-stimulating factor (GM-CSF). Standards and samples were diluted (1:4) in sample diluent and transferred to the plate containing magnetic beads for 1 h at RT. Next, the plate was washed (3×) and the detection antibody was added for 30 min on a shaker (850 rpm) at RT. After that, the plate was washed (3×) and streptavidin-PE solution was added for 10 min. Subsequently, the plate was washed (3×) and the samples were re-suspended in 125 µL of assay buffer and analysed within 15 min. All samples were analysed at the same time in duplicates. All reagents and technology were provided by Bio-Rad Laboratories (Bio-Plex 200).

### 4.4. Statistics

Results were analysed using Bio-Plex Manager 5.0 software. All results are given as an arithmetic means ± SD from the experiments; the number of repetitions varied depending on the type of experiment. Analyses of WJ-MSC secretome were conducted in 4 repetitions; for WJ-MSC + MS CSF we managed 11 independent experiments; for WJ-MSC + control CSF we managed 7 independent experiments. The part of the study with PMA addition was managed as 2 independent experiments for the WJ-MSC cultures, 3 experiments for the WJ-MSC + MS CSF, and 2 experiments for the WJ-MSC + control CSF. In experiments with the WJ-MSCs and MO3.13 co-culture, data were given from 4 samples of WJ-MSCs; 3 samples for WJ-MSCs + MO3.13; 2 samples for WJ-MSCs with PMA and 3 samples for WJ-MSCs + MO3.13 with PMA. For cell cultures of WJ-MSCs and CSFa, statistical significance was determined by Student’s *t*-test.

## 5. Conclusions

The use of MSCs in MS therapy raises high hopes among patients and researchers. WJ-MSCs have become possible sources of stem cells with a great potential of immunomodulatory activity. Studies with WJ-MSCs conditioned with human CSF revealed a lower proliferation rate but promotes the differentiation into neural-like cells. Our data showed a rich Secretome in WJ-MSCs with markedly increased levels of IL-6, IL-8, IP-10 and MCP-1 secretion and a favourable profile of growth factors. The addition of MS CSF to the WJ-MSC culture caused depletion of most measured factors, only IL-12, RANTES and GM-CSF levels were increased. Most cytokines and chemokines decreased their concentrations in the WJ-MSCs co-culture with the oligodendrocyte precursor cell line regardless of the maturation status; the levels of only eotaxin and RANTES were slightly increased. These results emphasize the spectrum of the immunomodulatory properties of WJ-MSCs and show how this effect can be modulated depending on the transplantation milieu.

## Figures and Tables

**Table 1:** Changes in factors secreted by WJ-MSCs growing in CSF and oligendrocytic MO3.13 cell line microenvironment. Most of the secreted cytokines, chemokines and growth factors concentrations were reduced by conditioning with CSF or MO3.13 cell line co-culture; increased factors are listed in the table.

Culture Type	Increased Secretion
WJ-MSCs + MS CSF v. WJ-MSCs	Il-12, RANTES, GM-CSF
WJ-MSCs + control CSF v. WJ-MSCs	-
WJ-MSCs + PMA v. WJ-MSCs	Il-5, Il-6
WJ-MSCs + PMA + MS CSF v. WJ-MSCs + MS CSF	IL-1b, IL-1ra, IL-4, IL-5, IL-6, IL-7, IL-8, IL-9, IL-17, IFN-γ, eotaxin, MCP-1, RANTES; G-CSF, VEGF
WJ-MSCs + PMA + control CSF v. WJ-MSCs + control CSF	IL-1b, IL-6, IL-8, IFN-γ, eotaxin and RANTES; G-CSF
WJ-MSCs + MO3.13 v. WJ-MSCs	Eotaxin, RANTES
WJ-MSCs + PMA + MO3.13 v. WJ-MSCs + MO3.13	MIP-1α, RANTES

## Data Availability

The data presented in this study are available on request from the corresponding author.
